# Validation of a Remote Sampling Sensor for Measuring Urine Volume and Nitrogen Concentration in Grazing Dairy Cattle

**DOI:** 10.3390/ani14202977

**Published:** 2024-10-15

**Authors:** Mancoba C. Mangwe, Nigel Beale, Paige Beckett, Lucas Tey, Jeffery Curtis, Riki Burgess, Racheal H. Bryant

**Affiliations:** Faculty of Agriculture and Life Sciences, Lincoln University, Lincoln 7647, New Zealand; mancoba.mangwe@lincoln.ac.nz (M.C.M.); nigel.beale@lincoln.ac.nz (N.B.); paige.fisher@lincoln.ac.nz (P.B.); lucas.tey@lincoln.ac.nz (L.T.); jeffery.curtis@lincoln.ac.nz (J.C.); riki.burgess@lincoln.ac.nz (R.B.)

**Keywords:** *Lolium perenne*, *Medicago sativa*, salt, urination behavior monitoring, urine nitrogen excretion

## Abstract

The purpose of this research was to validate a urine sensor (Lincoln University PEETER V2.0, Canterbury, New Zealand) that records the time and volume of urination events for dairy cows in addition to collecting a proportional urine sample from all urination events. Sixteen multiparous Holstein × Jersey mid-lactating cows (101 ± 5 days in milk, 498 ± 24.2 kg body weight, 26.2 ± 3.07 kg/d milk yield; mean ± standard deviation) were allocated herbage diets ranging in protein and sodium content to generate a range of urine volumes and urine nitrogen (UN) concentrations. Total collection of individual urination events occurred during a 72-h measurement period where PEETER V2.0 sensors were attached to cows. A mixed model ANOVA using lme4 package (version 1.1-35.5) in R (version 4.3.3) were used to compare the means. The average urine event size was 2.65 ± 1.1 L for total collection by observers and 2.68 ± 1.1 L as recorded by the sensor (mean ± standard deviation; *p* = 0.730). The urine nitrogen concentration was 5.76 ± 1.2 g N/L for samples collected by observers and 5.85 ± 1.3 g N/L for the samples collected by the sensor (*p* = 0.583). The calculated UN excretion was 156 ± 45.1 g/day for direct measurements and 162 ± 40.0 g/day for the sensor (*p* = 0.539. Contrasts with simultaneously measured data were undertaken using Lin’s Concordance Correlation Coefficient (CCC) and a Pearson correlation coefficient (r). Correlations between the actual values and sensor values were strong, with little to moderate variability in the urine volume (CCC = 0.936, r = 0.937; *n* = 222), UN concentration (CCC = 0.840, r = 0.837, *n* = 48) and total UN excretion (CCC = 0.827, r = 0.836, *n* = 24). Considering the findings, the PEETER V2.0 urine sensor has the potential to reliably measure urine volumes and UN concentrations for estimations of the UN excretion of dairy cattle under grazing systems.

## Simple Summary

The need for suitable tools to measure urination behavior in the field during pastoral grazing is well recognized. The current study investigated the accuracy of a modified urine sensor (PEETER V2.0) that measures urine volume and collects a proportional urine sample from every urination event. These can then be used for analysis of the urine nitrogen concentration and an estimation of the daily urine nitrogen excretion of each cow. The findings indicate a strong and positive correlation between the actual and sensor-derived values regarding urine volume. There was also a strong and positive correlation between the samples collected by the sensor and observers regarding urine nitrogen concentration and the calculated urinary nitrogen excretion. Further analysis indicated that the nitrogen concentration of the urine sample collected by the sensor is a reasonable mean of the daily average urinary nitrogen concentration. Therefore, the sensor is a real-time tool that measures urine volume and collects a sample for analyzing and estimating the mean urinary N excretion from grazing dairy cattle. This could be useful for evaluating the impacts of various strategies, such as nutritional manipulation, on urinary nitrogen excretion in grazing systems in the field.

## 1. Introduction

Urinary nitrogen (UN) excretion is an important component of nutrient recycling in pastoral grazing systems; however, at high nitrogen (N)-loading rates it is also associated with several environmental polluters, such as nitrate leaching to freshwater [1,2] and nitrous oxide emissions [3]. Consequently, many mitigation strategies to attenuate the environmental effects of intensive ruminant production systems have focused on altering UN excretion [4]. The approaches vary between manipulating the diet to reduce the total UN excretion (g N/day, [5]) and altering the distribution of individual urine events (g N/event, [6,7]). Thus, for robust modeling of N losses in pasture-based systems and for the development of strategies to diminish N losses, there is a requirement to accurately measure the temporal distribution and total UN excretion of individual cows [8,9].

There are limited methods for measuring the voluntary urination behavior [urine volume per event (L/event) and UN concentrations (g N/L)] in grazing systems. Although N balance studies carried out using animals in metabolism stalls provide accuracy, the cut and carry nature of this approach is unable to reflect the impact of diet selection and diurnal N fluxes which occur in pastoral conditions. The need for suitable tools to measure urination behavior in the field is well recognized, and several urine sensors have been developed to automatically and remotely record the urination behavior characteristics of dairy cattle under field conditions [10,11,12]. Marshall et al. [13] recently validated an earlier model of the Lincoln University PEETER V1.0 (Canterbury, New Zealand) urine sensor which measures and records urine event sizes and the times of urinations. With the PEETER V1.0 sensor, the volume is determined by measuring “flow against time” to establish a net volume [14]. Whilst this is important, the sensor did not measure the UN concentration.

The AgResearch acoustic sensor (Hamilton, New Zealand), which attaches to the leg of the cow, measures and records the time, duration and frequency of urinations for each cow by identifying spectral acoustic patterns that have a urination event-like spectral signature [15]. Like the PEETER V1.0, the acoustic sensor does not include measurements of UN concentration. On the other hand, the AgResearch Mark II (Hamilton, New Zealand) urine sensor uses a refractive index and pressure sensor to evaluate the individual and daily urine volumes and UN excretions of lactating dairy cattle [7]. Because the refractive index measures the extent to which a beam of light bends when it enters a material, it is not compound specific and could be modified by variations in the composition of the urine [16]. The Lincoln University team has developed a sensor (PEETER V2.0) that has a small sample bottle attached at the base of the unit to collect proportional urine samples during each urination event. The collected urine sample can later be analyzed for chemical components of the urine, such as N, for UN excretion estimations. Acidifying urine samples during total collection or immediately after collection is regarded as the best method for preserving N during in vivo experiments to prevent volatilization [17]. The intention for the use of this sensor is to avoid the need for acids in the field. The urine entering the bottle is only in a low-oxygen environment for a short period of time (24 h) before processing.

The purpose of this research was to validate the PEETER V2.0 sensor, which records the time and volume of urination events of dairy cows in addition to collecting a proportional urine sample from all urination events.

## 2. Materials and Methods

### 2.1. Urine Sensor Design

**Figure 1:**
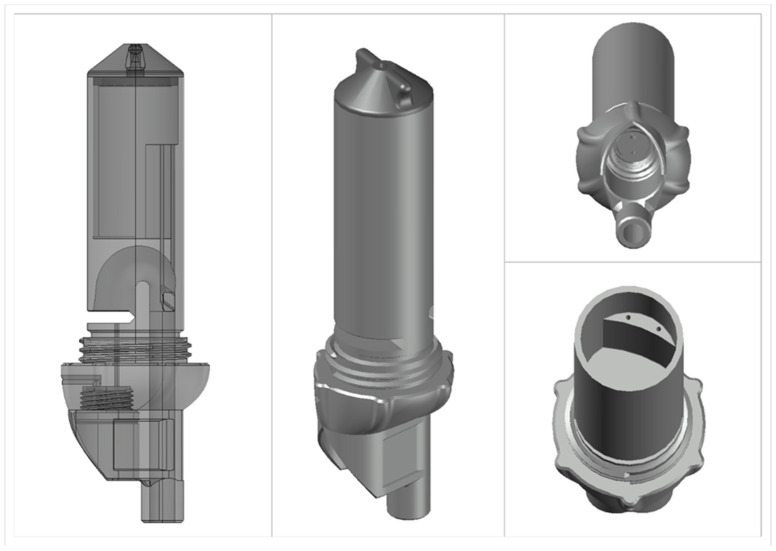
PEETER V2.0 urine sensor.

The PEETER V2.0 sensor (Figure 1) is a development of the PEETER V1.0 where identified issues from the PEETER V1.0 validation [13] have been addressed. Fused deposition modeling (FDM) was utilized, and additive manufacturing was chosen as the method of manufacturing. Additive manufacturing allows for an iterative design process, as the sensor helps in discovering performance-tested improvements that can be manufactured immediately in response to findings. Polyethylene Terephthalate Glycol (PETG) was chosen as the 3D printing material due to its durability, stability, and potential recyclable properties.

The basic principle of the sensor is to measure an area under the curve flow rate at 100 millisecond intervals to establish the net volume of each urination event. A 1psi pressure transducer (Honeywell, Digikey.co.nz (accessed 11 July 2024)) measures the head pressure via an Arduino microcontroller (Adafruit.com (accessed 11 July 2024), Adafruit, New York, NY, USA) and records the event, volume and timestamp to an SD card, which is retrieved when exchanging the sensor. From historical data and the trial feedback of the PEETER V1.0, the PEETER V2.0 has an improved flow path targeting 2 L to 5 L as the optimum range for an event while allowing for an accurate calibrated range of 1 L to 10 L.

The PEETER V2.0 sensor has a sample bottle attached to the base of the sensor. The sample bottle is filled proportionally to the flow as the fluid passes through the sensor via a small bypass tube at approximately 8 mL/L. In each sequential event, the bottle continues to receive a proportional sample, and the excess volume is passed out through an outlet hole. It is assumed that, because the proportional sampling is low, each sequential sample taken would result in a mean concentration of all the collected samples.

### 2.2. In Vitro Validation

Volume calibration was performed on a rig designed to mimic the height of the complete harness attached to a dairy cow. A bucket of 10 L of fluid placed above the rig was passed through the sensor system 4 times, each at a discreet height. A plot of the relationship of height and time for the given volume to pass calculated a best-fit polynomial curve, giving a height to flow velocity in L/millisecond. The calibration was verified by passing volumes of fluid through the sensor at 1, 2, 3, 4 and 10 L and repeating the event 4 times. The laboratory target accuracy was less than ±5% for 2 L and had a greater volume.

### 2.3. In Vivo Validation

#### 2.3.1. Experimental Design and Treatments

The validation with dairy cows was undertaken at the Ashley Dene Development Research Station in Springston, Canterbury, New Zealand (43°38′49.73″ S, 172°20′45.78″ E) between November and December 2023 with permission from the Lincoln University Animal Ethics Committee (#2023–56). Sixteen Holstein Friesian × Jersey mid-lactating cows used for the validation experiment were balanced into four groups of four cows per group based on days in milk (101 ± 5 days in milk), body weight (498 ± 24.2 kg), and milk yield (26.2 ± 3.07 kg/d). To test the sensors under a wide range of urine volumes and UN excretion groups, the four groups of cows were randomly allocated to one of four diets: (a) a ryegrass-white clover mix (PRW) with 200 g/cow/day of salt, (b) a PRW without salt, (c) a PRW plus lucerne with 200 g of salt/cow/day and (d) a PRW plus lucerne without salt supplementation.

#### 2.3.2. Adaptation Phase

Cows were acclimatized to diets over ten days under grazing conditions and adapted to metabolism crates over four days prior to measurements. While grazing, lucerne cows grazed pure lucerne swards between morning and afternoon milking and PRW swards between afternoon and the following morning milking. Cows on PRW-based diets were offered fresh PRW swards following each milking session. All cows grazed PRW herbage together in 1 ha paddocks after afternoon milking.

When cows were restrained in metabolism crates, milking was undertaken using a portable milking machine (DeLaval vacuum pump DVP170; DeLaval: Tumba, Sweden) twice daily at 6:30 a.m. and 3:00 p.m. Forage was cut twice daily at 6 a.m. and 2 p.m. and fed fresh after each of the two milking sessions. The forage was harvested 4 cm above ground using a 3-point linkage UFO twin drum mower (Aitchison, Palmerston North, New Zealand). Lucerne herbage was mixed with PRW herbage in equal proportions during each of the feeding sessions. The amount of feed required per cow was determined based on observations during the four-day crate-acclimatization period. Each cow was offered ad libitum feed to allow for approximately 10% refusals. The mean daily dry matter intake averaged 19.1 ± 2.7 kg/cow. The salt was sprinkled on top of the fresh feed in the bins during the morning and afternoon feeding sessions.

#### 2.3.3. Validation Phase

**Figure 2:**
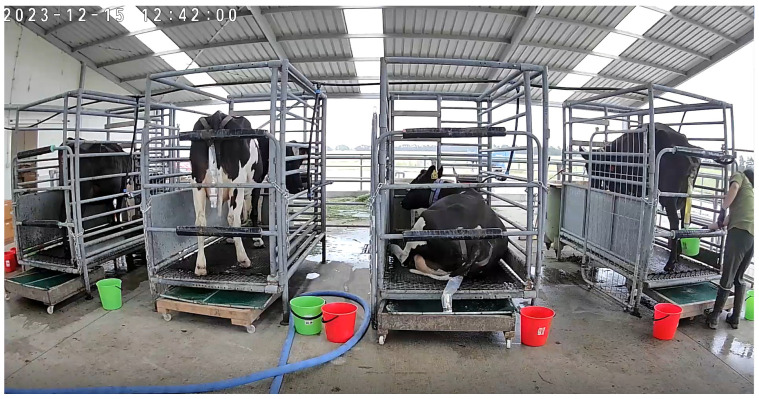
Set up and attachment of sensors to animals during the validation experiment.

The validation of the sensor was determined over four runs of 4 cows per run × 72-h period (16 cows × 72 h = 1152 h of real-time observation), whereby one cow represented each diet during each run. The metabolism crates (1.95 m long × 2.2 m high and 1.2 m wide) contained rubber mats that were fixed to the floor of the crates, and the cows were able to sit and stand but were unable to turnaround (Figure 2). Each metabolism crate had individual feed bins and automatic water troughs.

A harness was used to attach the sensor to the animals. The assembly of the harness and its placement over the vulva of the cow has been described in previous publications [10,14]. Briefly, the harness consists of a vinyl fabric (Zephyr Vinyl; Spotlight, South Melbourne, Australia), a ventilated 3D-printed mold (PETG) and a 40 cm plastic sleeve (Shardlows Packaging Ltd., Christchurch, New Zealand). The sensor attaches at the bottom of the sleeve through a locking mechanism secured with zip ties and a water sealant. The harness and sensor unit weighs <500 g. The unit was attached to the animal over the vulva using a strong adhesive (Loctite 454; Henkel, Düsseldorf, Germany). All urine flows through the sensor, which then records the volume and time of each urination event in addition to collecting a proportional urine sample from all the urination events. At the end of each 24-h period, the urine sampling bottle from the sensor was removed and replaced with a clean empty bottle. The sample collected by the sensor was split into 2 equal subsamples: one half was acidified with sulphuric acid and the other half was left untreated. After subsampling, all urine samples were immediately stored at −20 °C until analysis of the N concentration by Elementar (Vario MAX CN, Elementar Analysensysteme, Hanau, Germany).

The total urine was collected in real-time by 8 trained observers in 8-h shifts involving two trained personnel during the 72-h measurement period when cows were restrained in crates (Figure 2). The observation commenced at 9 a.m. on the first day, when the sensors were attached, until 9 a.m. on the fourth day of the measurement period. Prior to the commencement of the experiment, the total collection and recording procedures of urination behaviors were agreed upon on site by 9 observers and 3 experienced technicians. Each observer had a 15-min break after 2 h of continuous observation. At each urination event, observers placed a bucket underneath the harness and the sensor and collected all the urine. Beneath each crate was a collection tray for capturing any urine that was missed by observers. Urine in the collection tray was collected into a second bucket for determining the total volume. After each urination event, the time and weight of the urine (kg) were recorded—to two decimal places—using a portable scale. These were then compared with the sensor-derived values for urine volume for each urination. A subsample of urine was extracted from the clean bucket sample and decanted into two 25 mL vials, with one vial being immediately acidified with approx. 2 drops of sulphuric acid, and the other vial remaining unacidified. The subsamples were later analyzed for N concentration as described above. A urine sample was not collected from the urine captured in the tray as it was contaminated with fecal matter. Defecations, when they occurred, were captured in a net over the collection tray, which was kept clean by observers.

Two 24-h video surveillance cameras (TP-Link Tapo C110 Home Security Wi-fi Camera and TP-Link Tapo Outdoor Pan & Tilt WiFi Security Camera, Melbourne, Australia) were used to cross-check the sampling procedures. At the end of the experiment, the video footage was reviewed for each urination event as recorded by the sensor and by the observers.

### 2.4. Statistical Analysis

All statistical analyses were undertaken within R software, Version 4.2.0 [18]. A mixed models ANOVA using a lme4 package (version 1.1-35.5) was used to compare the means of the direct observers and the sensor, with the sampling method being used as a fixed effect and the data nested in the run, plus the salt nested in the forage type, being used as random effects. The accuracy of the sensor for all parameters, including urine volume, UN concentration and total UN excretion, was assessed in three steps. First, a scatter plot was created using the “ggplot2” package (version 3.5.1) within the tidyverse [19] suite of packages. A regression model was fitted using the “lm” function to the paired data, with direct measurements acting as the independent variable and the sensor-derived values acting as the dependent variable. Secondly, a Pearson correlation was calculated via the “cor.test” function in R to establish the strength and level of significance of the association between the paired observations. The Pearson correlation coefficient (r) determines how far each observation varied from the best-fit line [20]. Thirdly, the Lin’s Concordance Correlation Coefficient (CCC; [21]) was determined using the “CCC” function of the DescTools package (version 1.0) in R. The CCC indicates how close the two observations are to one another. It is calculated as a product of the Pearson correlation coefficient (a precision measure) and bias correction factor (C_b_; an accuracy measure). Other outputs from a typical call of “CCC” function include scale shift (*v*), which is a measure of the ratio of standard deviations of the two techniques whereby a value of 1 implies that the two techniques have similar standard deviations. The other output is the location shift (*u*), which is a measure of the variation between the means relative to scale (mean bias). In line with the Pearson correlation coefficient, the Lin’s CCC is expressed as a value between −1 and 1 where −1 represents perfect disagreement, 0 represents no agreement, and 1 represents perfect agreement [22]. The CCC can be interpreted in line with other correlation coefficients, such as the Pearson correlations coefficient, where values ranging from 0.00 to 0.30 are considered negligible, 0.31 to 0.50 slight, 0.51 to 0.70 minor, 0.71 to 0.90 moderate, and 0.91 to 1.00 strong [23,24].

The accuracy of the N concentration from the samples collected by the sensor was first ascertained by cross-checking that there was no N loss in the sampling bottle (urine acidification measurements). Nitrogen loss may occur during urine collection or storage because of ammonia volatilization and urea decomposition [17]. Urea and ammonia account for the majority of N in the urine [25]. Several studies [17,26] have suggested the use of either sulphuric or hydrochloric acids to preserve ammonia in the urine. Secondly, we checked whether the N concentration in the bottle represented the volume-adjusted N concentration measured for individual events. Urinary N concentration results for acidified samples were plotted against non-acidified sensor bottle samples (*n* = 48). A regression model was fitted to the paired data, with acidified samples acting as the independent variable and non-acidified samples acting as the dependent variable. To ascertain that the N concentration in the sensor bottle accurately represented the average N concentration over all individual events, a volume-adjusted UN concentration of individual urination events collected by observers (*n* = 578) was used to calculate the mean UN concentration for each cow per day (*n* = 48). We then plotted the N concentration results for non-acidified samples collected by observers and those collected by the sensor, fitting a regression model to the paired data, as discussed above.

To evaluate the accuracy of urine mixing in the sensor bottle during urinations, an additional analysis was undertaken for UN concentration. The volume-adjusted UN concentration for individual events that occurred during the first, middle and final third of the total volume collected was determined. The N concentration in the 24-h sample was compared with the N concentration in each of the first, middle and final thirds of the total volume for each cow each day.

The daily UN excretion for the sensor was calculated by multiplying the N concentration (g N/L) of the sample collected after 24 h by the sensor-recorded daily urine volume (L/day; *n* = 24). The daily UN excretion for direct measurements was calculated by multiplying the volume-adjusted mean UN concentration of the direct measurements by the actual measured daily urine volume (L/day). The accuracy of the sensor for total UN excretion was ascertained by comparing the paired daily UN excretion (g/day) from each cow with the sensor and the direct measurement using the methods described in above sections.

## 3. Results and Discussion

### 3.1. Urine Sensor Performance

A total of 578 individual urine samples were collected by observers during the validation experiment, though the sensor recorded 271 urinations (47% success rate). An analysis of the sensors identified two issues with the sensor variant that was in use during the trial. The first issue was air leaks allowing slugs of urine inside the pressure sensor’s air tubes, which caused the sensor to stop recording. This issue has been addressed by moving the air tubes to new locations and adding S/S 1.6 mm of needle tubing. The second issue was urine leaking into electronics when collecting the SD card. This was addressed with additional protection of the electronics and by adding cleaning steps to the standard operating procedure. Although the modified sensor did not make it into the trial, laboratory testing of the modified sensor in comparison with the trial variant showed these modifications did not alter the core function, design or working accuracy (see Table S1 and Figure S1).

Of the 271 sensor-recorded events, a complete set of 24-h data from the sensor (providing daily urine volumes per cow) was collected from 12 cows, 4 of which provided a single set of 24-h data whilst the rest provided a set of 24-h data for two (*n* = 4) or three (*n* = 4) consecutive days (24 observations). Occasionally, direct observers would miss whole or partial samples when multiple cows urinated/defecated at the same time. The use of 24-h surveillance of the pens enabled the removal of data that represented human errors (*n* = 49). Thus, 222 paired observations concurrently recorded by the sensor and observers were used for urine volume validation.

Despite the sensor not consistently recording the time and volume of every urination event, the bottles continued to collect samples over the full 72-h period. These were subsequently used for N concentration validation. As a result, there was a 100% success rate for the sample pottle, with 48 samples being collected from the sensor. The 578 samples collected by observers and analyzed for N concentration were used to calculate the mean UN concentration of each cow per day, providing 48 UN concentration observations that matched samples collected by the sensor. As there were 24 observations for daily urine volumes from the sensor, we were able to estimate the total UN excretion per cow per day on 24 occasions. Descriptive and comparative results for the urine volume, UN concentration (g N/L), and UN excretion (g/day) are presented in the following sections.

### 3.2. In Vivo Validation

Urine volume per event, UN concentrations, the total N output means and confidence intervals are displayed in Table 1. The average urine volume per event was similar for direct measurements and sensor-derived values (2.64 ± 0.26 L/event; mean ± standard error of the mean; *p* = 0.730) The range between the 0.37 and 9.56 L/event obtained in the current experiment is within the range of 0.30 to 9.5 L/event reported for cows in a study with the total collection coming from metabolism crates feeding PRW with or without increasing proportions of high-moisture forage plantains (*Plantago lanceolata* L. [27]). The current mean event size of 2.7 L/event is consistent with the mean event size of 2.5 L/event reported in a study with the total collection from metabolism crates feeding PRW silage with 0, 200 or 400 g salt to non-lactating dairy cows [28]. It is also consistent with the mean event size of 2.4 L/event determined using the AgResearch Mark II urine sensor for late-lactating cows grazing diverse swards containing high-moisture herbs [29]. The results confirm the sensor as a reliable measure of urine volume, which is consistent with the previous validation report of the PEETER V1.0 [13].

Table 2 displays the Pearson correlation coefficient, CCC, the mean bias presented as C_b_, the location shift presented as *u* and the scale shift presented as *v*. The Pearson correlation coefficient of 0.937 (*p* < 0.001) and the Lin’s CCC value of 0.936 of direct measurements and sensor recordings indicate strong and positive correlations with slight variations between methods. The mean bias was very low, indicating a mean difference of 0.025 L. The ratio of standard deviation of the sensor recordings to that of direct measurements of 1 indicates identical standard deviations for the two methods. The strength of the relationship is further demonstrated by the high r-squared value of 0.88 displayed in Figure S2. The points were evenly distributed along the regression line, confirming the strong and positive correlation, with some variability occurring between the two methods. The results confirm the Lincoln University PEETER V2.0 urine sensor as a reliable measurer of urine volume, which is consistent with the previous validation report of the PEETER V1.0 [13].

Marshall et al. [13] reported a slightly weaker correlation between direct measurements and the PEETER V1.0 sensor (R^2^ = 0.81 and CCC = 0.90) compared to the current results of the PEETER V2.0 (R^2^ = 0.88 and CCC = 0.94; Table 2). The urine flow rate was slower in the PEETER V1.0 sensor, which lead to urine overflows in the sensor sleeves when the sleeve was filled urine. PEETER V2.0 has an improved siphon design which restricts the flow targeting 2 to 5 L as the optimum range for a dairy cow event without overflowing. This appears to have improved the relationship between the direct measurements and the sensor recordings.

**Figure 3:**
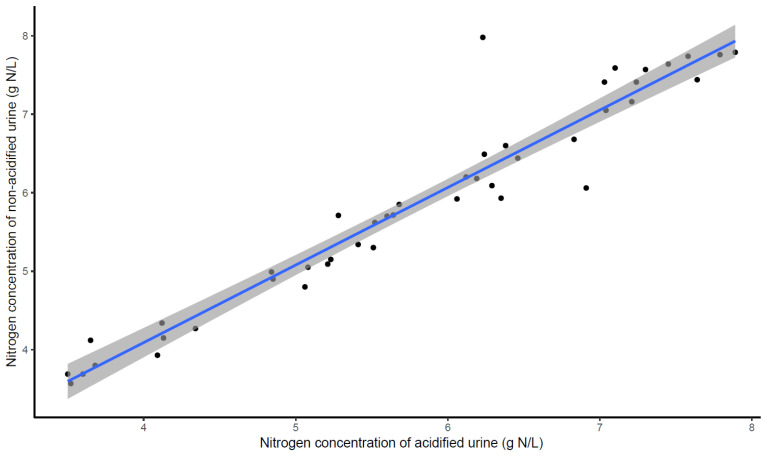
The regression line comparing the urinary nitrogen (UN) concentration (g N/L) of PEETER’s V2.0 acidified and non-acidified urine samples. Each back dot represents one paired UN concentration. The blue solid line is the regression line (y = 0.141 + 0.988x, R^2^ = 0.93), with the 95% CI being shown by the shaded band. Each data point represents one paired UN concentration.

The method of processing urine samples post collection did not influence the concentration of N, as evidenced by the similarities in the means and standard deviations (5.79 ± 1.13 versus 5.88 ± 1.33 g N/L; *p* = 0.586) of acidified and non-acidified urine samples collected by the sensor. The association between acidified and non-acidified samples was strong, with an R^2^ = 0.94 (Figure 3). However, Figure 3 displayed two points falling off the regression line, which likely reflects human error in sample mixing and/or contamination. Nonetheless, there was a uniform distribution of points along the regression line, suggesting that the acid and non-acid samples resulted in similar UN concentrations. The Pearson correlation coefficient (r = 0.964; *p* < 0.001) and CCC value (0.963) further demonstrated strong and positive correlations, with slight variations occurring between the acidified and non-acidified samples for N concentration.

The mean UN concentration was similar (5.81 g N/L; *p* = 0.583; ranged between 3.57 and 8.18 g N/L) for samples collected by observers compared to those collected by the sensor (Table 1). The relationship of the concentration of N in urine samples collected by the sensor and the direct measurements was strong and positive with moderate variations (r = 0.840; *p* < 0.001 and CCC = 0.837; Table 2). The relationship between samples collected by observers compared with samples collected by the sensor for UN concentration is further displayed in Figure S3, showing a strong correlation and an R^2^ value of 0.73. The mean and range of UN concentration obtained in the current experiment are consistent with the mean range of 3.6 to 7.9 g N/L determined using the AgResearch Mark II urine sensors [12] and the mean range of 4.3–7.5 g N/L reported for mid-lactating cows in metabolism crates fed freshly cut PRW only or PRW with increasing proportions of high-moisture forage herbs such as plantains and chicory (*Cichorium intybus* L. [30]).

**Figure 4:**
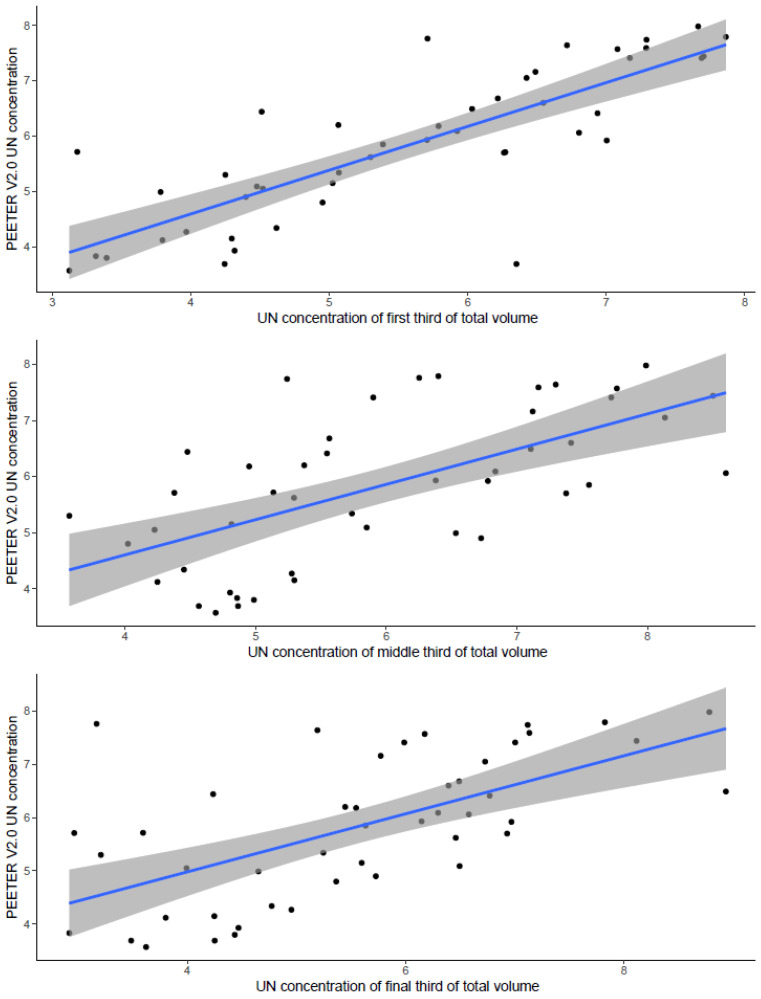
Regression analysis comparing the urine nitrogen (UN) concentration (g N/L) from the PEETER V2.0 urine sensors and the first, middle and final third of the actual urine volume. Each back dot represents one paired UN concentration. The blue solid line is the regression line (first third; y = 1.43 + 0.791x, R^2^ = 0.67; second third; y = 2.09 + 0.629x, R^2^ = 0.40; y = 2.80 + 0.545x, R^2^ = 0.39), with the 95% confidence interval being shown by the shaded band.

Comparing the PEETER V2.0 urine from the sensor bottle sample with observer- measured urine during either the first, middle, or final third of the daily urine volume in this study reduced the strength of the relationship for UN concentration (Figure 4). The total urine volume per subsection was on average 9.73 L (range: 6.26 to 15.2 L) for the first, 9.19 L (range: 4.32 to 14.3 L) for the middle, and 10.1 L (range 5.9 to 20.0 L) for the final third of the daily urine volume. The mean UN concentration was 5.6 g N/L (range = 3.1 to 7.7 g N/L) for the first, 5.9 g N/L (range 3.6 to 8.5 g N/L) for the middle third, and 5.6 g N/L (range 2.9 to 8.7 g N/L) for the final third of the daily urine volume. The strength of the relationship was low (first, R^2^ = 0.67; middle; R^2^ = 0.40; and final third, R^2^ = 0.39) compared to the overall UN concentration (Figure S3, R^2^ = 0.73). The outliers become more dominant with fewer samples in the subsection analysis. This indicates that the mean urine N concentration in the sensor bottle is a reasonable mean of the entire collection period.

The calculated total UN excretion averaged 156 g/day (ranging from 80 to 267 g/day) for direct measurements and was similar (*p* = 0.539: Table 1) to the 162 g/day (ranging from 93 to 236 g/day) calculated based on the sensor-collected samples. The overall mean total UN excretion of both methods (159 ± 9.49 g N/day) is well within the range of UN excreted by Northwestern Europe (mean = 162, range = 64 to 346 g/day), North America (mean = 200, range = 61 to 366 g/day) or New Zealand lactating dairy cattle (mean = 176, range = 66 to 365) [31]. The samples collected by the sensor had a strong and positive correlation with moderate variations compared with those collected by direct measurements (r = 0.836; *p* < 0.001 and CCC = 0.827; Table 2). The relationship between the actual values and the values obtained by the PEETER V2.0 sensor regarding the estimated UN excretion levels displayed in Figure S4 shows a strong correlation and an R^2^ value of 0.70.

## 4. Conclusions

The findings show that there is a strong and positive relationship between direct measurements and the Lincoln University PEETER V2.0 sensor in regard to urine volume, UN concentration, and the UN excretion of dairy cows. Therefore, the PEETER V2.0 sensor is a real-time tool that can remotely measure urination behavior and enable the calculation of UN excretion from grazing dairy cattle. This could be useful for evaluating the impacts of various strategies, such as nutritional manipulation, on UN excretion measurements from pastoral systems in the field.

## Figures and Tables

**Table 1:** Urine volume per event (L/event), urine nitrogen concentration (g N/L) and total nitrogen concentration (g N/day) from direct observations compared with PEETER V2.0 recordings/samples (mean ± standard deviation).

		Direct Measurements		PEETER V2.0			
Item	*n*	Mean	95% CI ^1^	Mean	95% CI ^1^	SEM	*p* Value
Urine volume (L/event)	222	2.65 ± 1.05	1.85–3.45	2.68 ± 1.06	1.88–3.48	0.26	0.730
Urine nitrogen concentration (g N/L)	48	5.76 ± 1.18	4.87–6.66	5.85 ± 1.33	4.96–6.74	0.30	0.583
Total nitrogen excretion (g N/day)	24	156 ± 45.8	132–181	162 ± 40.5	138–186	9.49	0.539

^1^ CI = confidence interval, SEM = standard error of the mean.

**Table 2:** Findings of the in vivo validation experiment with the Pearson correlation coefficient (r), and Lin’s Concordance Correlation Coefficient (CCC) of actual measurements compared with PEETER V2.0 recordings and measurements.

Item	Correlation (r)	*p* Value	Bias Correction Factor (C_b_)	CCC ^1^	Location Shift (*u*)	Scale Shift (*v*)
Urine volume (L/event)	0.937	<0.0001	0.999	0.936	0.025	1.00
Urine nitrogen concentration (g N/L)	0.840	<0.0001	0.994	0.837	0.055	1.10
Total nitrogen excretion (g N/day)	0.836	<0.0001	0.99	0.827	−0.076	1.13

^1^ CCC = Lin’s Concordance Correlation Coefficient.

## Data Availability

All data used in the experiment are presented in the Supplementary Materials.

## Supplementary Materials

The following supporting information can be downloaded at https://www.mdpi.com/article/10.3390/ani14202977/s1, Table S1. Descriptive and comparative statistics of target water volume (L) and sensor water volumes during laboratory validation of the modified PEETER V2.0. Figure S1. Laboratory validation of the modified PEETER V2.0 urine sensor for urine volume measurements. The blue line is the regression line (y = 0.129 + 0.954x, R2 = 1.00). Each data point represents one paired observation (*n* = 200). Figure S2. Regression of urine volume per event (L/event) recorded by the PEETER V2.0 sensor and direct measurement. The blue line is the regression line (y = 0.178 + 0.937x, R2 = 0.88), with the 95% CI being shown by the shaded band. Each data point represents one paired urination event. Figure S3. Regression analysis comparing the urine nitrogen concentration (g N/L) from PEETER V2.0 urine sensors and direct measurements. The blue solid line is the regression line (y = 0.495 + 0.926x, R2 = 0.71), with the 95% confidence interval being shown by the shaded band. Figure S4. Regression analysis comparing urine nitrogen excretion (g/day) from the PEETER V2.0 urine sensors and direct measurements. The blue solid line is the regression line (y = 42.0 + 0.739x, R2 = 0.70), with the 95% confidence being interval shown by the shaded band. Raw Data for Analyses: Table S2: Description tab for items used in raw data tables. Table S3. Sensor versus target volume during laboratory validation of the modified PEETER sensor. Table S4. Direct-measured urine volume versus sensor-recorded urine volume–271 observations. Table S5. Direct-measured urine volume versus sensor-recorded urine volume–222 observations. Table S6. Urine nitrogen concentration of acidified and non-acidified urine samples. Table S7. Urine nitrogen concentration of the three subsections of daily urine volume and urine nitrogen excretion from observers and the sensors.
