# Supplementary material for: Validation of a Remote Sampling Sensor for Measuring Urine Volume and Nitrogen Concentration in Grazing Dairy Cattle
Source: Animals (Basel). 2024 Oct 15;14(20):2977. doi: 10.3390/ani14202977 (PMC11503686; doi:10.3390/ani14202977)
Supplement: Supplementary file 1 [file animals-14-02977-s001.zip › animals-3127338-supplementary.pdf]

Supplementary Materials and Raw Data Used for Analyses

TableS1. Descriptive and comparative statistics of target water volume (L) and device water volumes during laboratory validation of modified PEETER V2.0.

Static	Target volume		Modified PEETER V2.0 urine sensors
**Descriptive**			
Number of observations	200		200
Mean	4.29		4.22
Standard deviation	2.67		2.55
Maximum value	10.1		9.87
Minimum value	0.975		1.00
**Comparative[1]**			
Pearson's correlation coefficient (r)		1.00	
Bias correction factor (C_b)		1.00	
Concordance correlation coefficient (CCC)		0.998	
Location shift (*u*)		-0.025	
Scale shift (*v*)		0.955	

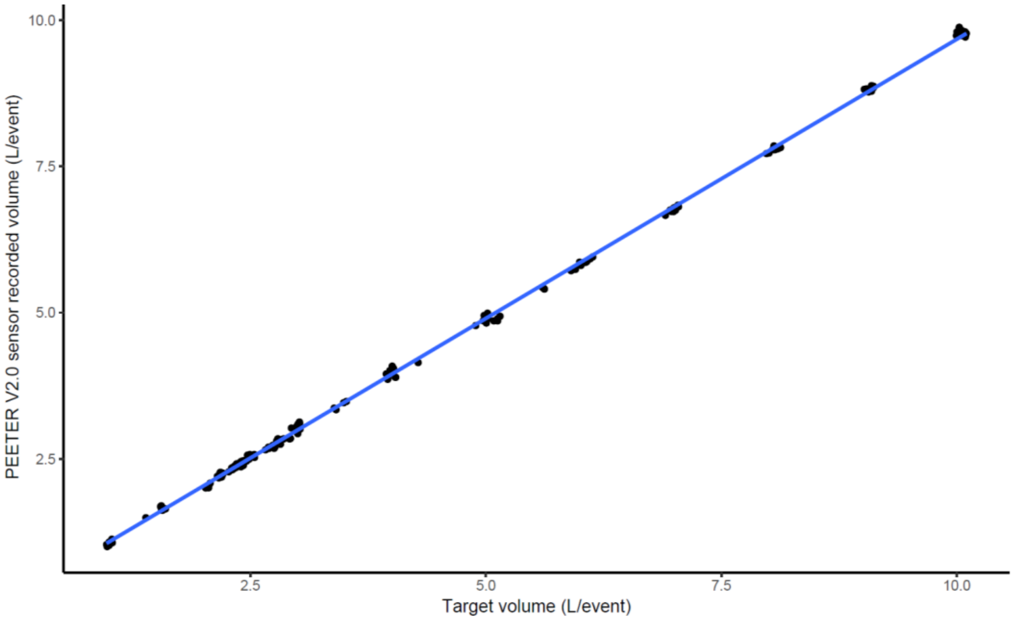

Figure S1. Laboratory validation of the modified PEETER V2.0 urine sensor for urine volume measurements. The blue line is the regression line (y = 0.129 + 0.954x, R^2 = 1.00). Each data point represents one paired observation (*n* = 200).

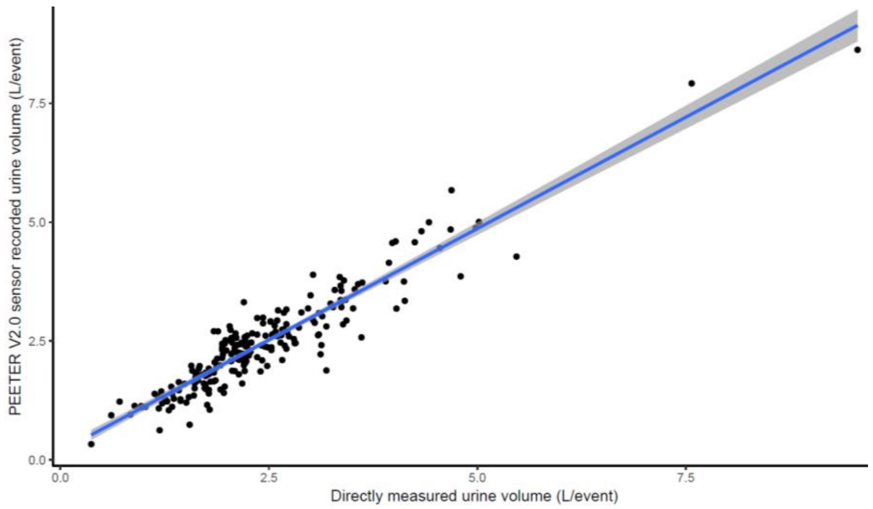

Figure S2. Regression of urine volume per event (L/event) recorded by the PEETER V2.0 sensor and direct measurement. The blue line is the regression line (y = 0.178 + 0.937x, R^2 = 0.88), with the 95% CI shown by the shaded band. Each data point represents one paired urination event.

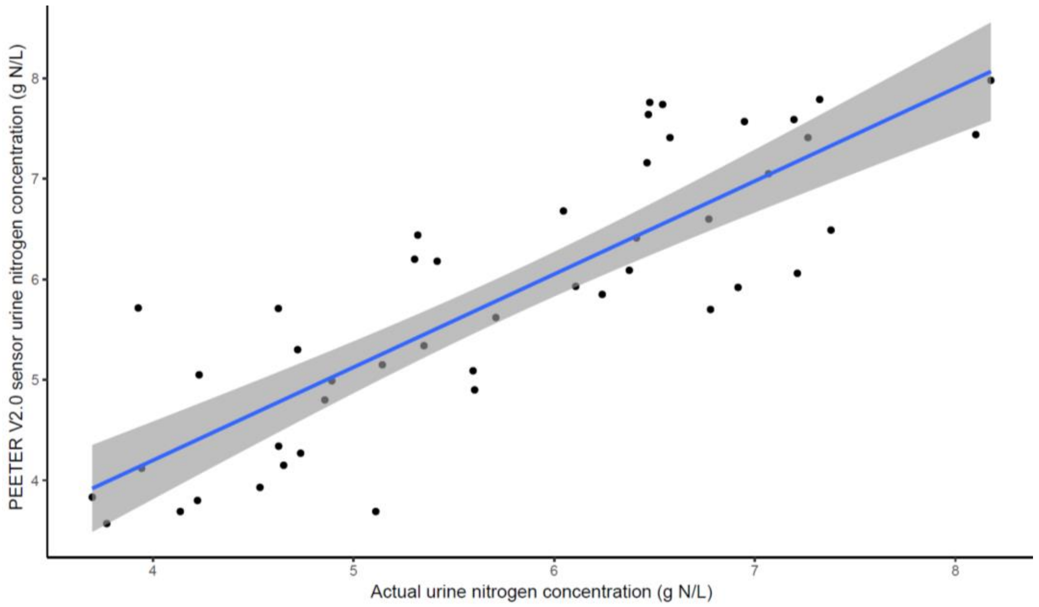

Figure S3. Regression analysis comparing urine nitrogen concentration (g N/L) from PEETER V2.0 urine sensors and direct measurements. The blue solid line is the regression line (y = 0.495 + 0.926x, R^2 = 0.73), with the 95% confidence interval shown by the shaded band.

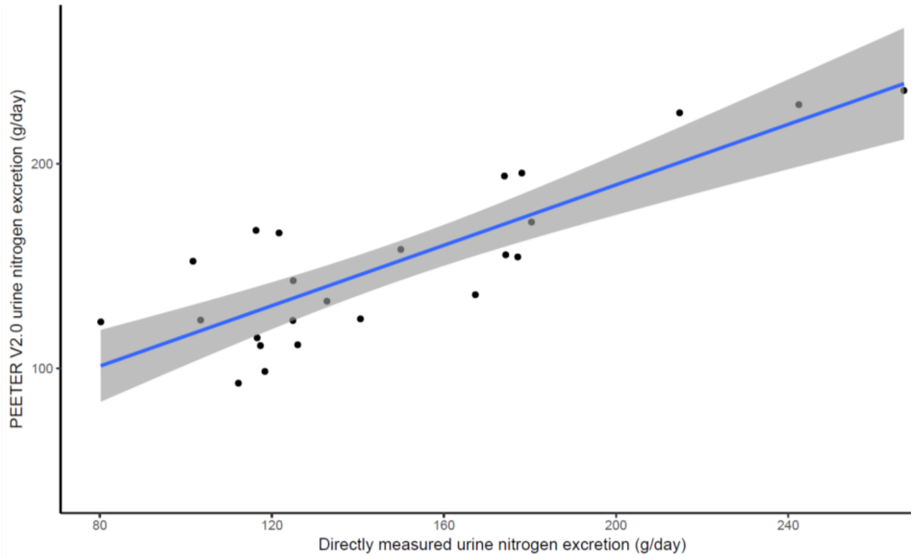

Figure S4. Regression analysis comparing urine nitrogen excretion (g/day) from PEETER V2.0 urine sensors and direct measurements. The blue solid line is the regression line ($y = 42.0 + 0.739x$, $R^2 = 0.70$), with the 95% confidence interval shown by the shaded band.

Raw Data for Analyses

Table S2: Description tab for items used in raw data tables.

**Item**	**Description**	**Units**
Sensor.Volume	Sensor volume during modified PEETER laboratory validation	L/event
Target Volume	Target volume during modified PEETER laboratory validation	L/event
Cow	Cow identity number	
ObserversV1	Urine volume recorded by observers before viewing	L/event
SensorV1	Urine volume recorded by urine sensor	L/event
Volume1-271Obs	Urine volume before removing 49 events due to human error	L/event
Volume2-222Obs	Urine volume after removing 49 events due to human error	L/event
Non acidified	Non acidified sensor urine samples	g/L
Acidified	Acidified urine sensor samples	g/L
BlockANcon	Urine nitrogen concentration for first third of daily urine volume	g/L
BlockBNcon	Urine nitrogen concentration for middle third of daily urine volume	g/L
BlockCNcon	Urine nitrogen concentration for final third of daily urine volume	g/L
ObserverNCon	Volume adjusted mean urine N concentration	g/L
SensorN.output.noacid	Urine sensor-based N excretion	g/day
ObserverNexc	Direct measurements-based N excretion	g/day

Table S3. Sensor versus target volume during laboratory validation of modified PEETER sensor

Sensor.Volume	Target Volume	Sensor.Volume	Target Volume
1.07	1.04	3.04	3.00
1.08	1.03	4.01	3.98
1.13	1.03	4.03	4.00
1.09	1.02	4.05	4.01
2.40	2.43	4.04	4.01
2.37	2.40	4.05	4.02
2.39	2.43	3.99	3.99
2.39	2.40	3.95	3.94
3.37	3.39	4.09	4.01
3.48	3.52	4.05	4.01
3.46	3.49	4.03	4.00
3.34	3.41	4.99	5.02
4.94	5.15	4.95	4.98
4.86	5.09	4.95	5.01
4.86	5.13	4.82	5.01
4.90	5.13	4.86	4.99
9.80	10.00	4.91	5.03
9.73	10.00	4.86	4.97
9.88	10.03	4.88	5.01
9.76	10.02	4.89	5.00
1.02	1.00	4.78	4.89
1.00	0.98	5.92	6.11
1.08	1.00	5.86	6.00
1.04	0.98	5.95	6.14
2.09	2.07	5.74	5.95
2.01	2.06	5.88	6.07
2.01	2.03	5.85	6.03
2.06	2.06	5.81	6.01
2.85	2.93	5.72	5.91
2.84	2.92	5.86	6.06
2.97	3.00	5.86	6.05
2.93	3.00	6.75	7.00
3.86	3.96	6.79	6.99
3.88	3.96	6.73	6.99
3.90	4.04	6.75	7.01
3.90	4.04	6.73	6.97
4.15	4.28	6.81	7.05
5.42	5.61	6.66	6.91
5.40	5.62	6.80	7.01
5.43	5.60	6.84	7.04
3.01	3.03	6.75	6.96
3.05	3.03	7.82	8.13
3.06	3.00	7.83	8.12
3.07	3.02	7.80	8.10
3.07	3.01	7.73	8.01
3.11	3.02	7.81	8.11
3.13	3.02	7.81	8.06
3.10	3.01	7.78	8.07
3.03	2.94	7.72	7.98

Table S4. Direct measured urine volume versus sensor recorded urine volume – 271 observations.

Cow	ObserversV1	SensorV1	Cow	ObserversV1	SensorV1
297	1.74	1.581	55	4.25	4.577
297	1.38	1.822	55	3.29	3.572
297	2.14	2.107	55	3.98	4.562
297	1.65	1.691	55	2.43	2.872
297	2.14	1.798	55	1.19	1.831
297	1.34	2.144	55	3.03	2.361
297	1.94	1.389	55	1.13	2.731
297	2.13	2.858	55	4.13	3.344
297	2.21	2.58	55	2.29	2.468
297	1.3	3.571	55	3.16	4.584
297	0.71	1.224	55	1.57	1.979
297	2.66	2.099	55	3.14	3.02
297	2.36	2.983	55	3.03	2.94
297	1.51	1.205	55	1.67	1.972
297	1.92	1.987	55	4.8	3.86
297	2.45	2.384	55	2.22	2.045
297	2.57	2.824	160	1.44	1.24
297	1.94	3.893	160	2.28	2.274
297	3.11	4.278	160	2.66	2.738
297	2.59	3.684	160	1.57	2.603
297	1.81	2.891	160	1.24	2.649
297	1.78	1.473	186	1.36	1.286
297	1.67	1.784	186	2.2	1.99
297	3.36	3.362	186	1.18	1.861
297	3.1	2.637	186	2.48	1.975
297	2.67	2.702	186	1.18	1.081
297	2.04	2.735	186	1.57	0.899
297	2.37	2.924	186	3.35	1.539
297	1.75	1.78	186	2	3.025
297	2.24	2.215	186	2.52	2.914
297	2.24	2.374	186	2.1	2.659
297	2.26	2.195	186	2.6	2.932
55	1.58	0.165	186	3.26	3.948
55	0.89	1.134	186	0.83	3.192
55	2.4	1.86	186	1.87	0.314
55	2.48	2.37	186	3.19	1.88
55	1.8	1.646	186	1.78	1.396
55	1.61	1.504	186	2.68	3.094
55	1.26	2.125	186	1.42	1.463
55	2.3	2.661	186	2.31	1.587
55	2.45	3.074	186	2.24	2.459
55	2.6	2.6	186	1.66	2.64
55	6.81	5.464	186	1.98	2.518
55	1.78	1.919	186	2.47	3.433

55	1.89	1.98	530	3.05	2.882
530	2.15	2.25	470	1.17	1.331
530	2.71	3.162	470	0.9	1.587
530	3.09	2.621	470	3	3.46
530	2.43	2.99	470	4.24	2.596
530	3.62	3.729	470	3.61	1.789
530	3.94	4.145	470	1.54	1.32
530	3.35	3.846	470	1.44	1.274
530	3.1	3.087	470	5.07	3.772
530	2.52	2.906	470	1.6	1.353
530	3.36	3.663	470	1.86	1.671
530	2.73	3.397	470	1.97	1.54
530	2.2	3.316	470	0.97	1.1
530	3.57	2.458	470	1.72	1.608
530	2.46	3.578	470	3.12	2.22
530	3.24	3.286	470	2.41	1.653
530	2.89	3.1	470	1.19	0.623
530	2.97	3.184	470	0.84	0.949
530	2.11	2.568	470	1.55	0.739
530	1.99	2.301	470	1.3	1.043
530	2.71	2.852	470	2.53	2.309
530	2.68	2.741	470	1.73	1.508
530	1.76	2.547	470	3.51	3.185
530	1.25	2.444	470	2.46	2.611
530	2.05	2.563	470	1.34	1.116
530	2.21	2.139	470	1.74	1.766
530	3.19	2.807	470	1.3	2.483
279	1.94	2.317	470	1.6	1.641
279	2.21	2.492	470	1.22	1.189
279	2.14	2.185	490	2.17	2.463
279	1.94	2.442	490	2.39	0.864
279	1.06	1.829	490	2.42	2.093
279	1.71	1.793	490	0.61	0.938
279	2.79	3.378	490	1.78	1.802
279	1.56	2.211	490	2.15	2.171
279	2.16	2.434	490	2.69	2.404
279	2.06	2.467	490	1.74	1.832
279	2.23	2.077	490	1.28	1.229
279	2.41	2.408	490	1.79	1.056
279	2.12	2.417	490	1.84	1.835
279	1.89	2.703	490	1.21	1.439
279	1.47	2.98	490	2.81	2.452
279	2.52	4.852	490	1.27	2.011
279	1.97	2.921	490	1.13	1.389
279	1.59	3.23	490	1.75	1.483
279	2.03	2.809	490	1.58	1.877
279	1.88	2.127	490	1.26	1.272
279	2.03	2.511	490	2.18	1.606
279	2.24	3.441	490	1.96	1.408

470	1.02	1.113	490	1.92	1.479
470	0.37	0.33	490	4.42	4.997
470	1.85	2.053	490	0.97	1.131
470	1.61	1.612	490	1.76	1.156
490	2.64	2.467	321	1.65	1.669
490	2.21	1.868	321	2.06	1.874
490	2.05	2.079	321	2.12	2.127
490	2.22	2.217	321	2.61	3.144
490	2.71	2.336	321	1.02	1.677
490	1.64	1.67	321	1.97	2.443
490	2.52	3.881	321	1.62	1.729
490	2.07	2.571	321	2.12	2.262
490	2.36	2.159	321	1.87	1.941
490	1.84	2.705	321	2.54	2.637
361	3.43	2.93	321	1.42	1.635
361	3.9	3.755	321	1.84	1.651
361	3.37	3.211	321	1.33	1.537
361	3.13	2.413	321	2.05	2.225
361	3.27	3.211	321	2.09	2.358
361	3.37	3.329	321	2.14	2.11
361	2.86	2.783	94	2.82	2.456
361	2.2	2.06	94	1.66	1.919
361	4.68	4.845	94	2.36	2.635
361	2.24	2.612	94	2.72	2.687
361	2.24	2.441	94	1.49	1.6
361	4.02	4.594	94	2.53	2.368
361	9.56	8.624	94	2.09	1.868
361	4.69	5.67	94	3.61	2.576
361	5.47	4.275	94	3.39	2.851
361	2.58	2.696	94	2.65	2.622
161	4.98	4.88	94	2.04	2.076
161	4.55	4.454	94	1.84	1.849
161	3.42	3.367	125	4.12	3.75
161	5.02	5.004	125	4.03	3.181
161	3.37	3.555	125	2.8	2.541
161	4.33	4.808	125	2.22	2.388
161	2.08	2.272	125	2.21	2.44
161	3.4	3.773	125	1.95	2.218
161	3.57	3.695	125	1.62	1.826
161	7.57	7.917	125	2.19	2.235
161	3.53	3.587	125	1.74	1.768
161	2.23	2.476	125	2.54	2.612
			125	2.74	2.598

Table S5. Direct measured urine volume versus sensor recorded urine volume – 222 observations.

Cow	ObserversV2	SensorV2	Cow	ObserversV2	SensorV2
55	0.89	1.134	470	3.12	2.22
55	2.4	1.86	470	1.19	0.623
55	2.48	2.37	490	1.58	1.877
55	1.8	1.646	490	1.26	1.272
55	1.61	1.504	490	2.18	1.606
55	2.3	2.661	490	1.96	1.408
55	2.6	2.6	490	1.92	1.479
55	1.78	1.919	490	4.42	4.997
160	1.44	1.24	490	0.97	1.131
160	2.28	2.274	530	3.35	3.846
160	2.66	2.738	530	3.1	3.087
186	1.36	1.286	530	2.52	2.906
186	2.2	1.99	530	3.36	3.663
186	2.48	1.975	530	2.2	3.316
186	1.18	1.081	530	3.24	3.286
186	2.52	2.914	279	1.88	2.127
186	2.1	2.659	279	2.03	2.511
186	2.6	2.932	470	0.84	0.949
297	1.74	1.581	470	1.55	0.739
297	2.14	2.107	470	1.3	1.043
297	1.65	1.691	470	2.53	2.309
297	2.14	1.798	470	1.73	1.508
297	1.34	1.389	470	3.51	3.185
297	1.94	2.144	470	2.46	2.611
297	2.21	2.58	470	1.34	1.116
297	0.71	1.224	470	1.74	1.766
55	1.89	1.98	470	1.6	1.641
55	4.25	4.577	470	1.22	1.189
55	3.29	3.572	490	1.76	1.156
55	3.98	4.562	490	2.64	2.467
55	2.43	2.872	490	2.21	1.868
55	3.03	3.893	490	2.05	2.079
186	3.19	1.88	490	2.22	2.217
186	1.78	1.396	490	2.71	2.336
186	2.68	3.094	490	1.64	1.67
186	1.42	1.463	490	2.07	2.571
186	2.24	2.459	490	2.36	2.159
186	1.98	2.518	490	1.84	2.705
186	2.47		530	2.89	3.1
297	2.66	2.099	530	2.97	3.184
297	2.36	2.983	530	2.11	2.568
297	1.51	1.205	530	1.99	2.301

297	1.92	1.987	530	2.71	2.852
297	2.45	2.384	530	2.68	2.741
297	2.57	2.824	530	2.05	2.563
297	1.94	2.361	530	2.21	2.139
297	1.78	1.473	530	3.19	2.807
55	4.13	3.344	161	4.98	4.88
55	2.29	2.468	161	4.55	4.454
55	1.57	1.979	321	1.65	1.669
55	3.14	3.02	321	2.06	1.874
55	3.03	2.94	321	2.12	2.127
55	1.67	1.972	321	2.61	3.144
55	4.8	3.86	321	1.97	2.443
55	2.22	2.045	321	1.62	1.729
297	1.67	1.784	321	2.12	2.262
297	3.36	3.362	321	1.87	1.941
297	3.1	2.637	321	2.54	2.637
297	2.67	2.702	321	1.42	1.635
297	2.04	2.735	321	1.84	1.651
297	1.75	1.78	361	3.43	2.93
297	2.24	2.215	361	3.9	3.755
297	2.24	2.374	161	3.42	3.367
297	2.26	2.195	161	5.02	5.004
279	1.94	2.317	161	3.37	3.555
279	2.21	2.492	161	4.33	4.808
279	2.14	2.185	161	2.08	2.272
279	1.94	2.442	161	3.4	3.773
279	1.71	1.793	161	3.57	3.695
470	1.02	1.113	161	7.57	7.917
470	0.37	0.33	321	1.33	1.537
470	1.85	2.053	321	2.05	2.225
470	1.61	1.612	321	2.09	2.358
470	1.17	1.331	321	2.14	2.11
470	3	3.46	361	3.37	3.211
470	1.54	1.32	361	3.13	2.413
470	1.44	1.274	361	3.27	3.211
490	2.17	2.463	361	3.37	3.329
490	2.42	2.093	361	2.86	2.783
490	0.61	0.938	361	2.2	2.06
490	1.78	1.802	361	4.68	4.845
490	2.15	2.171	361	2.24	2.612
490	2.69	2.404	161	3.53	3.587
490	1.74	1.832	161	2.23	2.476
490	1.28	1.229	361	2.24	2.441
490	1.79	1.056	361	4.02	4.594
490	1.84	1.835	361	9.56	8.624
490	1.21	1.439	361	4.69	5.67

490	2.81	2.452	361	5.47	4.275
490	1.13	1.389	361	2.58	2.696
490	1.75	1.483	125	4.12	3.75
530	3.05	2.882	125	4.03	3.181
530	2.15	2.25	125	2.8	2.541
530	2.71	3.162	125	2.22	2.388
530	3.09	2.621	125	2.21	2.44
530	2.43	2.99	125	1.95	2.218
530	3.62	3.729	125	1.62	1.826
530	3.94	4.145	125	2.19	2.235
279	2.16	2.434	125	1.74	1.768
279	2.06	2.467	125	2.54	2.612
279	2.23	2.077	125	2.74	2.598
279	2.41	2.408	94	2.82	2.456
279	2.12	2.417	94	1.66	1.919
279	1.89	2.703	94	2.36	2.635
279	2.03	2.809	94	2.72	2.687
470	1.6	1.353	94	1.49	1.6
470	1.86	1.671	94	2.53	2.368
470	1.97	1.54	94	2.09	1.868
470	0.97	1.1	94	3.61	2.576
470	1.72	1.608	94	3.39	2.851
94	2.04	2.076	94	2.65	2.622
94	1.84	1.849			

Table S6. Urine nitrogen concentration of acidified and non-acidified urine samples

Nonacidified	Acidified	Nonacidified	Acidified
9.00	9.15	3.80	3.68
8.27	8.68	6.18	6.19
5.30	5.51	4.34	4.12
6.44	6.46	4.12	3.65
6.06	6.91	7.76	7.79
7.16	7.21	5.92	6.06
3.69	3.50	4.27	4.34
4.99	4.84	3.93	4.09
4.90	4.85	6.09	6.29
5.85	5.68	5.70	5.60
3.83	3.83	4.80	5.06
5.72	5.64	5.05	5.08
7.41	7.24	7.05	7.04
6.20	6.12	7.74	7.58
7.64	7.45	6.68	6.83
3.57	3.52	5.93	6.35
7.41	7.03	7.79	7.89
5.34	5.41	6.60	6.38
5.71	5.28	6.49	6.24
3.69	3.60	5.09	5.21
7.57	7.30	7.98	6.23
4.15	4.13	7.59	7.10
7.44	7.64	5.62	5.52
5.15	5.23		

Table S7. Urine nitrogen concentration of the three subsections of daily urine volume and urine nitrogen excretion from observers and sensor.

BlockANcon	BlockBNcon	BlockCNcon	ObserverNCon	SensorN.output.noacid	ObserverNexc
5.88	5.48	5.84	5.73		184.67
5.38	5.55	5.74	5.87		210.39
4.25	3.57	3.21	4.72	166.22	121.66
4.51	4.48	4.24	5.32	152.40	101.66
6.80	8.60	6.58	7.21	155.53	174.35
6.49	7.12	5.77	6.46		202.54
6.35	4.86	4.25	5.11		137.88
3.78	6.53	4.65	4.89	158.14	149.96
4.40	6.73	5.73	5.60	154.49	177.15
5.39	7.55	5.63	6.24		216.26
3.32	4.86	2.92	3.70		112.85
3.18	5.14	3.59	3.93	167.48	116.31
7.17	7.72	7.00	7.27	195.48	178.10
5.07	5.37	5.45	5.30	123.41	124.88
6.72	7.30	5.19	6.47	224.93	214.77
3.12	4.69	3.62	3.77	132.85	132.79
7.69	5.90	5.99	6.58	235.85	266.91
5.07	5.74	5.25	5.35	98.52	118.37
6.27	4.38	2.96	4.63		146.34
4.24	4.56	3.49	4.14	111.54	126.00
7.08	7.77	6.17	6.95		242.08
4.30	5.30	4.25	4.65	92.79	112.21
6.94	5.55	6.77	6.41	194.05	174.05
3.39	4.99	4.43	4.22	111.13	117.33
5.79	4.95	5.55	5.42		195.21
4.62	4.45	4.77	4.63	114.96	116.57
3.80	4.25	3.80	3.94		190.49
5.71	6.25	3.17	6.48		227.80
7.00	6.78	6.97	6.92	228.94	242.49
3.97	5.28	4.95	4.74	123.62	103.43
4.32	4.80	4.47	4.53	124.20	140.58
5.92	6.83	6.30	6.37		181.61
6.26	7.38	6.93	6.78		227.78
4.95	4.02	5.36	4.86	122.73	80.23
4.52	4.23	3.99	4.23	142.92	124.96
6.43	8.13	6.73	7.07		199.04
7.29	5.24	7.12	6.54		162.47
6.22	5.56	6.49	6.05		139.54
5.71	6.38	6.15	6.11	171.54	180.35
7.86	6.40	7.83	7.32		203.59
6.55	7.41	6.39	6.77		174.65
6.03	7.11	8.93	7.38	136.03	167.30
4.48	5.85	6.49	5.60		156.95
7.66	7.99	8.78	8.18		230.40
7.29	7.16	7.13	7.19		195.92
5.30	5.29	6.46	5.71		139.24
5.02	4.82	5.60	5.14		125.65
7.70	8.50	8.12	8.10		216.31
